# HIV viral load testing and monitoring in Côte d’Ivoire: A survival analysis of viral load testing and suppression, and evaluation of adherence to national recommendations

**DOI:** 10.1371/journal.pgph.0001822

**Published:** 2023-09-14

**Authors:** Kathryn E. Kemper, Orvalho Augusto, Stephen Gloyd, Derick A. Akoku, Gbossouna Ouattara, Lucy A. Perrone, Paul Henri Assoa, Chantal Akoua-Koffi, Christiane Adje-Toure, Ahoua Koné

**Affiliations:** 1 Health Alliance International, Seattle, Washington, United States of America; 2 Department of Global Health, University of Washington, Seattle, Washington, United States of America; 3 Health Alliance International, Abidjan, Côte d’Ivoire; 4 International Training and Education Center for Health, Seattle, Washington, United States of America; 5 International Training and Education Center for Health, Abidjan, Côte d’Ivoire; 6 Université Alassane Ouattara, Bouaké, Côte d’Ivoire; 7 University Teaching Hospital Bouaké, Ministry of Health and Public Hygiene, Bouaké, Côte d’Ivoire; 8 Centers for Disease Control and Prevention, Abidjan, Côte d’Ivoire; Public Health Ontario, CANADA

## Abstract

Routine viral load (VL) monitoring is the standard of care in Côte d’Ivoire and allows for effective treatment guidance for people living with human immunodeficiency virus (HIV) to reach viral load suppression (VLS). For VL monitoring to be effective in reducing the impact of HIV, it must be provided in accordance with national guidance. This study aimed to evaluate VL testing, VLS rates and adherence to national guidance for VL testing using data collected from three national laboratories. We collected data on VL testing between 2015–2018 from OpenELIS (OE), an open-source electronic laboratory information system. We merged data by unique patient ID for patients (0–80 years old) who received multiple VL tests to calculate time between tests. We defined VLS as HIV RNA ≤1,000 copies/mL based on Côte d’Ivoire national and WHO guidance at the time of data collection. We used the Kaplan-Meier survival estimator to estimate time between ART (antiretroviral therapy) initiation and the first VL test, time between subsequent VL tests, and to estimate the proportion of people living with HIV (PLHIV) who were virally suppressed within 12 months of ART initiation. At the first documented VL test, 79.6% of patients were virally suppressed (95% CI: 78.9–80.3). Children under 15 were the least likely to be virally suppressed (55.2%, 95% CI: 51.5–58.8). The median time from ART initiation to the first VL sample collection for testing was 7.8 months (IQR:6.2–13.4). 72.4% of patients were virally suppressed within one year of treatment initiation (95% CI:71.5–73.3). Approximately 30% of patients received a second VL test during the 4-year study period. The median time between the first and second VL tests was 24.9 months (IQR: 4.7->40). Most PLHIV received their first VL test within the recommended 12 months of ART initiation but did not receive subsequent VL monitoring tests within the recommended time frame, reducing the benefits of VL monitoring. While VLS was fairly high, children were least likely to be virally suppressed. Our findings highlight the importance of regular VL monitoring after the first VL test, especially for children.

## Introduction

Implementation of routine viral load (VL) testing has become the standard of care in many Sub-Saharan African countries as a method of monitoring patient progression towards developing AIDS and increasing the proportion of people living with human immunodeficiency virus (HIV) who have achieved viral load suppression (VLS) [1]. Routine VL monitoring allows for effective treatment guidance for people living with HIV (PLHIV) to achieve VLS. When VLS is attained, the risk of opportunistic infection and risk of sexual and vertical HIV transmission are reduced significantly, patients’ health outcomes are improved, and risk of morbidity and mortality decreases significantly [2]. The number of PLHIV in Côte d’Ivoire is approximately 380,000, the second highest HIV burden in West Africa [3]. Viral load monitoring can help identify and link PLHIV with elevated viral loads to supportive care, including enhanced adherence counselling and immediate antiretroviral therapy (ART) regimen change to achieve VLS. Therefore, scale-up and promotion of routine HIV viral load testing is essential towards achieving HIV epidemic control in Cote d’Ivoire.

In 2015 Côte d’Ivoire launched the “Test and Start” strategy, as well as a nationally backed initiative to significantly scale-up VL testing capacity and routine patient monitoring processes in clinics run by the Ministère de la Santé et de l’Hygiène Publique (Ministry of Health and Public Hygiene, [MSHP]) [1, 4]. Despite significant progress since 2015, major gaps remain towards achievement of the UNAIDS 95-95-95 goals of: 95% all PLHIV diagnosed, 95% of diagnosed PLHIV on ART treatment, and 95% of PLHIV on ART virally suppressed. At the end of 2020 in Côte d’Ivoire, 77% of PLHIV knew their status, 74% of whom were on ART, and 61% of PLHIV on ART were virally suppressed [3]. Since 2015, Côte d’Ivoire has continued to update national HIV treatment guidelines including the frequency of VL testing among PLHIV, improve laboratory data collection systems, and implement workforce training to improve VL testing and monitoring [4].

Prior to 2009, the majority of Côte d’Ivoire’s 200+ public laboratories used paper-based registers and reports to collect and to report laboratory data to clinicians and national disease monitoring programs. Since 2009, the University of Washington International Training and Education Center for Health (UW I-TECH) has worked in partnership with the MSHP to implement a comprehensive set of activities aimed at strengthening the laboratory system including the sustainable development, expansion, and technical support of an open-source electronic laboratory information system (eLIS). This collaboration has addressed the need for a comprehensive, customizable, low cost, open source eLIS to serve the needs of the public health systems with initial attention to HIV patients. The MSHP’s Direction de l’Informatique et de l’Information (Directorate of Informatics and Information, [DIIS]) adopted OpenELIS (OE) as its preferred eLIS in 2015 resulting in a 5 year-long initiative to expand OE installation and user training nationwide [5]. In 2018, ITECH developed the viral load dashboard for the MSHP which summarizes aggregate data from all VL testing laboratories with data extracted from the OE systems at those laboratories [6, 7].

VL monitoring can play a substantial role in reducing the impact of HIV in Côte d’Ivoire, but VL testing must be accessible, reliable, and consistent. Through a collaboration between Health Alliance International (HAI) and the International Training and Education Center for Health (I-TECH)–and the MSHP, we evaluated VLS rates among patients with HIV-1 and adherence to national guidelines for VL testing and monitoring using data from OE collected from three national laboratories of Côte d’Ivoire.

## Materials and methods

### Study design, setting, and participants

This study was a retrospective analysis of secondary data from a laboratory information system. The data used were part of a HIV clinical care program from three laboratories managed by HAI in north-central and eastern regions of Côte d’Ivoire from July 2015 to November 2018. In 2018, these laboratories served 223 clinics across 17 districts in the central, north central, and northern regions of Côte d’Ivoire. The study population included patients from these facilities, aged 0–80 years who were diagnosed with HIV, receiving ART treatment, and received at least one VL test during the study period.

Exclusion criteria included: i) patients aged 0–80 years old who initiated ART prior to July 2015 (due to inability to assess timing between treatment starting date and VLS); ii) patients who initiated ART after May 2018 (because the last date for viral load tests in this dataset was November 2018, ART initiation after May 2018 would not have allowed for adequate time between ART initiation and the recommended 6-month testing guidelines); iii) patients with missing data on year of ART initiation. All laboratory data included in this analysis was collected at the time of test initiation and extracted from the OE database [5]. We extracted and analyzed the following variables: patient demographics, clinical and immunological data, including the patient’s date of birth, gender, health facility/ART clinic, ART initiation date, ART regimen, blood sample collection date, date sample was received at the lab, date of testing, date of test validation, and VL results.

### Key definitions and statistical analysis

#### Data management

We merged data for patients who received multiple VL tests by matching on a unique patient ID variable. Some patients had partial or incomplete ART initiation dates entered into the system, many of which were labeled as January 1 or included ‘XX’ in the day and month fields. To account for impartial ART dates, we used interval censoring.

#### Definitions

**Fig 1:**
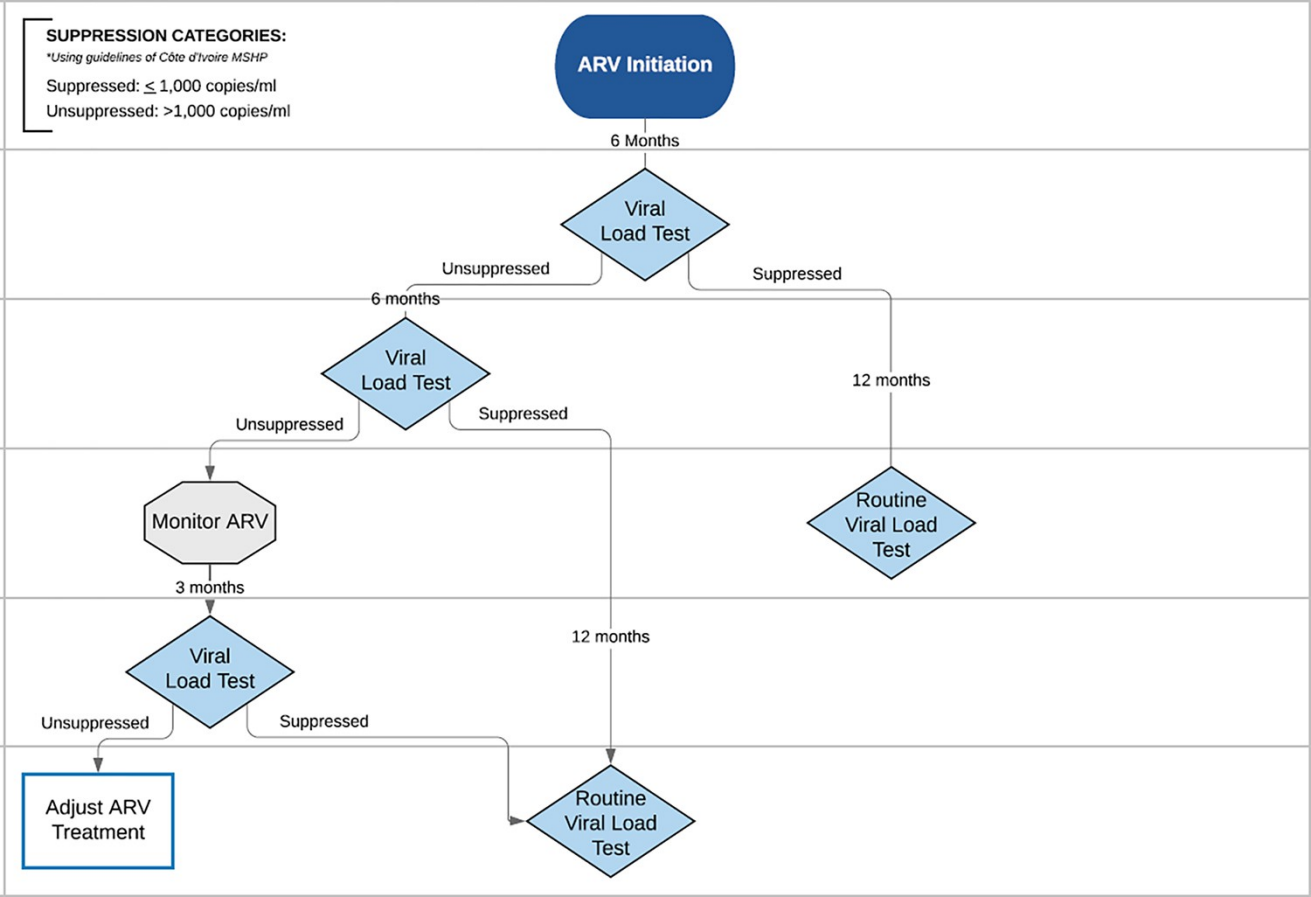
Côte d’Ivoire viral load testing algorithm based on MSHP guidance at data collection (2015–2018).

At the time of data collection and up until March 2021, the World Health Organization (WHO) defined viral suppression as HIV RNA ≤1,000 copies/mL of plasma [2, 8]. In March of 2021, WHO released updated recommendations on HIV viral load monitoring, reclassifying VLS as a viral load that is undetectable or ≤50 copies/mL [9]. For the purposes of this analysis, we created a binary variable for “suppressed,” classifying results of ≤1,000 copies/mL as virally suppressed and results of >1,000 copies/mL as “unsuppressed,” in accordance with national and WHO guidelines at the time of data collection. We defined VLS within 12 months as VLS resulting from the last test within 12 months post-ART initiation. To evaluate adherence to guidelines for VL monitoring, we considered a second VL test within 12 months of ART initiation as adherent for patients who were suppressed at their first VL test and a second VL test within 6 months of ART initiation for patients who were unsuppressed at their first VL test, per the national testing algorithm (Fig 1).

#### Statistical analysis

Summary statistics are presented as proportions for categorical variables and as medians with interquartile ranges (IQR) for continuous variables. For proportions, the exact 95% confidence intervals (95% CI) are presented and p-values from Pearson’s chi-squared tests are used to compare groups. Our primary outcomes were: 1) time between ART initiation and sample collection date of the first VL test; and 2) time between first and second sample collection date of VL monitoring tests. We used the Kaplan-Meier survival estimator to estimate time between ART initiation and sample collection dates of first VL test and between first and second VL tests. To compare the overall distribution of time (survival curve), a log rank test adapted for interval-censored data was used and p-values for median time duration comparison are presented. We used a Kaplan-Meier survival estimator to estimate the proportion of PLHIV who were virally suppressed within 12 months of starting ART. All data cleaning and analysis were conducted using R version 3.6.1 (The R Project for Statistical Computing) using the survival and interval libraries [10–12].

### Ethical approval and consent procedures

Ethical approval for this study was obtained from the Comité Consultatif National de Bioéthique de la République de Côte d’Ivoire and the University of Washington Institutional Review Board (IRB) and the Centers for Disease Control and Prevention (CDC) Division of Global HIV and TB (DGHT).

## Results

### Demographics and VLS at first test

A total of 12,467 HIV-infected patient records were included in this analysis. 72.8% were female, 94.1% were adults 15 years or older, and 5.9% were children under 15 (Table 1). We used this cut-off to correspond with Côte d’Ivoire’s national treatment guidelines at the time of data collection which defined children as under the age of 15. Most patients (71%) initiated ART in 2016 or 2017. Though all patients had the year of ART initiation documented, 73% did not have a complete ART initiation date, including day and month, entered into the OE system. At the first documented VL test, 79.6% of patients were virally suppressed (95% confidence interval [CI]: 78.9–80.3). Women were more likely to be virally suppressed (80.6% [95% CI: 79.8–81.4] compared to 76.8% of men [95% CI: 75.4–78.2], p<0.001). Children under 15 were the least likely to be virally suppressed (55.2% [95% CI: 51.5–58.8] compared to 72.8–87.4% for other age groups, p<0.001).

**Table 1:** Characteristics of patients who received viral load tests in Côte d’Ivoire, by viral load suppression at first test, 2015–2018.

	Total	Virally Suppressed at First VL test		
	N [%]	N [%]	(95% CI)	p-values
**Total**	12467 [100]	9922 [79.6]	(78.9–80.3)	
**Sociodemographics**				
Gender				<0.001
Male	3387 [27.2]	2602 [76.8]	(75.4–78.2)	
Female	9080 [72.8]	7320 [80.6]	(79.8–81.4)	
Age category (years)				<0.001
0–14	732 [5.9]	404 [55.2]	(51.5–58.8)	
15–24	933 [7.5]	679 [72.8]	(69.8–75.6)	
25–34	3535 [28.4]	2811 [79.5]	(78.2–80.8)	
35–49	5328 [42.7]	4365 [81.9]	(80.9–83.0)	
50–64	1749 [14.0]	1497 [85.6]	(83.9–87.2)	
65+	190 [1.5]	166 [87.4]	(81.8–91.7)	
Region where patient received ART*				<0.001
Bounkani-Gontougo	3453 [27.9]	2752 [79.7]	(78.3–81.0)	
Gbeke	4808 [38.9]	3952 [82.2]	(81.1–83.3)	
Hambol	1253 [10.1]	970 [77.4]	(75.0–79.7)	
Poro-Tchologo-Bagoue	2849 [23.0]	2175 [76.3]	(74.7–77.9)	
**Clinical Characteristics**				
Year of ART Initiation				<0.001
2015	1553 [12.5]	1180 [76.0]	(73.8–78.1)	
2016	4065 [32.6]	3298 [81.1]	(79.9–82.3)	
2017	4742 [38.0]	3785 [79.8]	(78.6–81.0)	
2018	2107 [16.9]	1659 [78.7]	(76.9–80.5)	
Location of central VL laboratory				<0.001
Abengourou	3453 [27.7]	2752 [79.7]	(78.3–81.0)	
Bouake	6808 [54.6]	5496 [80.7]	(79.8–81.7)	
Korhogo	2206 [17.7]	1674 [75.9]	(74.0–77.7)	

* 4 missing data observations

### Time to first VL test

The median time from ART initiation to the first VL sample collection was 7.8 months (IQR: 6.2–13.4) (Table 2). There were no significant differences in median time between ART initiation and first VL sample collection by patients’ age (p = 0.715) or gender (p = 0.266). Patients who initiated ART in 2015 did not receive a VL test for a median time of 20.6 months (IQR: 15.8–28.4) compared to patients who initiated ART in 2016 or later (median range: 4.8–11.5) (p<0.001).

**Table 2:** Estimated median time (in months) from ART initiation to first VL test among PLHIV who received a viral load test in Côte d’Ivoire, 2015–2018.

		Time (months) between treatment initiation and first VL test		
	N	Median (IQR)*	p-value Logrank^†^	p-value median**
**Total**	12,467	7.8 (6.2–13.4)		
**Sociodemographics**				
Age category (years)			0.025	0.715
0–14	732	8.2 (6.2–13.6)		
15–24	933	7.4 (5.7–11.9)		
25–34	3535	7.8 (6.0–13.3)		
35–49	5328	7.8 (6.2–13.6)		
50–64	1749	7.8 (6.2–13.7)		
65+	190	8.2 (6.1–12.2)		
Gender			0.008	0.266
Male	3387	7.6 (6.2–13.1)		
Female	9080	7.9 (6.1–13.5)		
Region			<0.001	<0.001
Bounkani-Gontougo	3453	7.5 (6.1–11.3)		
Gbeke	4808	8.9 (6.3–15.7)		
Hambol	1253	12.0 (7.1–18.8)		
Poro-Tchologo-Bagoue	2849	6.7 (5.5–9.0)		
**Clinical Characteristics**				
Year of ART Initiation			<0.001	<0.001
2015	1553	20.6 (15.8–28.4)		
2016	4065	11.5 (7.5–16.8)		
2017	4742	7.1 (6.1–8.8)		
2018	2107	4.8 (1.8–5.7)		
Lab			<0.001	<0.001
Abengourou	3453	7.5 (6.1–11.3)		
Bouake	6808	9.9 (6.5–16.6)		
Korhogo	2206	6.4 (5.4–7.6)		

* The median and quartiles of time are estimated through Kaplan-Meier survival curve to account for censoring in the time estimation

^†^ p-value computed from logrank test. This test compares the overall survival curve between groups.

** p-value to compare only the median survival times

### VLS within 12 months of ART initiation

We analyzed medical records for 10,360 patients who initiated ART at least 12 months prior to the last VL sample collection date and therefore had a full year of data post-ART initiation (Table 3). Of these patients, 68.3% reached VLS within one year of treatment initiation (95% CI: 67.3–69.3). There were no significant differences between males and females (68.5% [95% CI: 66.5–70.4] vs. 68.3 [95% CI: 67.1–69.4], respectively). We saw significant differences in VLS within 12 months based on year of ART initiation. Among patients who initiated ART in 2015, 21.8% were estimated to reach VLS in 12 months (95% CI: 19.7–23.9) compared to 62.0% and 95.7% of patients who initiated ART in 2016 and 2017 (95% CI: 60.3–63.5 and 94.8–96.4, respectively). Children under the age of 15 were the least likely age group to reach VLS after 12 months on ART– 57.6% were estimated to be virally suppressed after 12 months (95% CI: 52.9–61.8) compared to 68.4–72.3% among all other age groups.

**Table 3:** Proportion of patients estimated to reach VLS within 12 months among PLHIV who received a viral load test in Côte d’Ivoire, 2015–2018.

		VLS within 12 months	
	N	Est. Proportion (%)	95% CI
**Total** *	10360	68.3	(67.3–69.3)
**Sociodemographics**			
Age category (years)			
0–14	667	57.6	(52.9–61.8)
15–24	783	69.5	(65.5–72.9)
25–34	2928	68.7	(66.8–70.5)
35–49	4377	68.4	(66.9–69.8)
50–64	1446	70.6	(68.0–73.0)
65+	159	72.3	(63.9–78.8)
Gender			
Male	2729	68.5	(66.5–70.4)
Female	7631	68.3	(67.1–69.4)
Region			
Bounkani-Gontougou	2488	72.8	(70.8–74.7)
Gbeke	4457	64.4	(62.9–65.9)
Hambol	1174	53.8	(50.7–56.7)
Poro-Tchologo-Bagoue	2140	82.7	(80.7–84.6)
**Clinical Characteristics**			
Year of ART Initiation			
2015	1553	21.8	(19.7–23.9)
2016	4065	62.0	(60.3–63.5)
2017	4742	95.7	(94.8–96.4)
2018*	-	-	-
Lab			
Abengourou	2488	72.8	(70.8–74.7)
Bouake	6388	61.7	(60.4–62.9)
Korhogo	1484	97.4	(95.4–98.6)

*Table 3 excludes patients who initiated ART in 2018 as 12-month data was not available.

### Time to second VL test

3,704 (29.7%) of patients received a second VL test during the 4-year study period (Table 4). There were no significant differences within age (p = 0.091) or gender (p = 0.823) of patients who received a second VL test. More patients who were suppressed at their first VL test received a test compared to those unsuppressed at their first test (30.1% compared to 28.3%, p<0.001). Patients who initiated ART in 2016 were more likely to receive a second VL test compared to patients who initiated ART in any other year (39.4% compared to 4.5–33.0%, p<0.001). Children under the age of 15 were slightly less likely than other age groups to receive a second VL test (27.0% compared to 28.1–32.1%) and had a slightly longer median time estimate between the two tests (28.7 months compared to 23.2–25.7 months), but these differences were not statistically significant (p = 0.091).

**Table 4:** Estimated median time (in months) between first and second viral load tests among PLHIV who received a viral load test in Côte d’Ivoire, 2015–2018.

	Total	Patients who received a 2^nd^ VL test	Time (months) between first and second VL tests		
	N	N [%]	Median (IQR)*	p-value Logrank^†^	p-value median**
**Total**	12467	3704 [29.7]	24.9 (14.7 - >40)		
**Demographics**					
Age category (years)				0.227	0.091
0–14	732	198 [27.0]	28.7 (15.8 - >40)		
15–24	933	268 [28.7]	24.7 (14.2 - >40)		
25–34	3535	994 [28.1]	25.7 (14.9 - >40)		
35–49	5328	1625 [30.5]	25.0 (14.6 - >40)		
50–64	1749	562 [32.1]	23.4 (14.6–39.5)		
65+	190	57 [30.0]	23.2 (16.2 - >40)		
Gender				0.675	0.823
Male	3387	994 [29.3]	24.8 (14.8 - >40)		
Female	9080	2710 [29.8]	25.0 (14.7 - >40)		
Region				<0.001	<0.001
Bounkani Gontougou	3453	978 [28.3]	20.9 (13.9 - >40)		
Gbeke	4808	1496 [31.1]	27.3 (15.5 - >40)		
Hambol	1253	344 [27.5]	36.7 (20.6 - >40)		
Poro Tchologo Bagoue	2849	880 [30.9]	21.6 (13.6–26.2)		
**Clinical Characteristics**					
Results of first VL test				<0.001	<0.001
Suppressed	9922	2983 [30.1]	23.9 (14.4 - >40)		
Unsuppressed	2545	721 [28.3]	26.8 (15.9 - >40)		
Year of ART Initiation				<0.001	-
2015	1553	513 [33.0]	38.1 (25.5 - >40)		
2016	4065	1601 [39.4]	22.4 (14.9 - >40)		
2017	4742	1496 [31.5]	21.3 (13.2 - >40)		
2018	2107	94 [4.5]	NA		
Lab				<0.001	<0.001
Abengourou	3453	978 [28.3]	20.9 (13.9 - >40)		
Bouake	6808	2149 [31.6]	26.8 (16.6 - >40)		
Korhogo	2206	577 [26.2]	20.8 (13.4 - >40)		

* The median and quartiles of time are estimated through Kaplan-Meier survival curve to account for censoring in the time estimation

** p-value to compare only the median survival times

† p-value computed from logrank test. Notice this test compares the overall survival curve between groups.

The median time between the first and second VL tests was 24.9 months (IQR: 14.7->40). This time was shorter among patients who were virally suppressed at their first test (23.9 months compared to 26.8 months, p <0.001).

**Fig 2:**
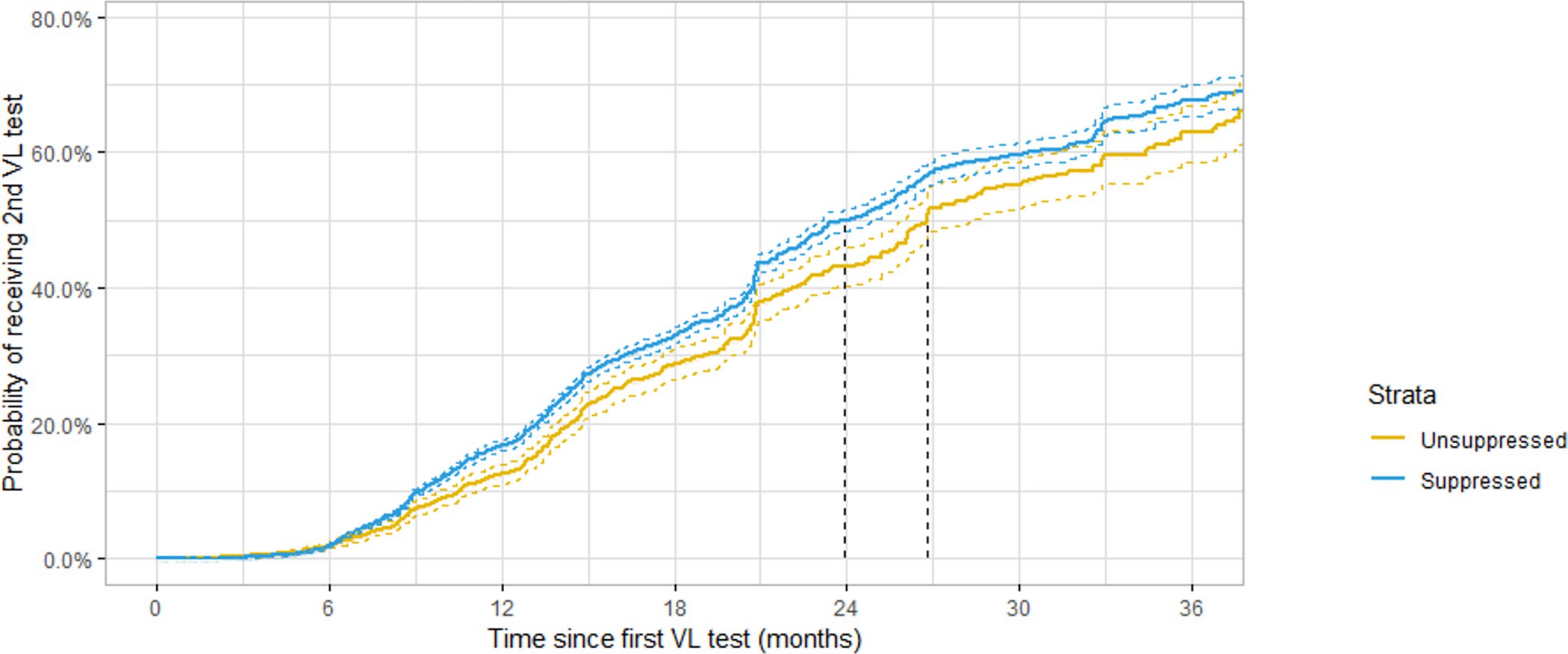
Kaplan-Meier survival curve for time between 1^st^ and 2^nd^ VL tests. Dotted lines represent median time in months between 1^st^ and 2^nd^ VL test for suppressed (23.9 months) and unsuppressed (26.8 months) patients, respectively.

Among the 2,545 patients who were not virally suppressed at their first VL test, the cumulative incidence of receiving a second VL test within 6 and within 12 months was 2% and 12.6%, respectively (Fig 2, Table 5). Among patients who were virally suppressed at their first VL test, cumulative incidence of receiving a second VL test within 6 months and within 12 months was 2% and 16.8%, respectively (Fig 2, Table 6). Fig 2 represents a Kaplan-Meier survival curve for the median time between 1^st^ and 2^nd^ VL tests, comparing patients who were virally suppressed at their first test and patients who were not virally suppressed at their first test. Virally suppressed patients had a 2^nd^ VL test after approximately 23.9 months, compared to 26.8 months for unsuppressed patients, despite national recommendations for a shorter testing interval of 6 months for virally unsuppressed patients.

**Table 5:** Survival estimates of time from first to second VL test among PLHIV who are virally unsuppressed at first VL test.

Time	Risk [n]	Event [n]	Cumulative Incidence (95% CI)
0 months	2545	0	0.0
3 months	2505	12	0.5 (0.2–0.7)
6 months	2372	37	2.0 (1.4–2.5)
12 months	1468	211	12.6 (11.1–14.0)
15 months	1081	158	22.7 (20.7–24.6)
18 months	817	78	28.8 (26.5–30.9)
21 months	491	85	37.9 (35.2–40.5)
24 months	373	38	43.2 (40.2–46.0)

**Table 6:** Survival estimates of time from first to second VL test among PLHIV who were virally suppressed at first VL test.

Time	Risk [n]	Event [n]	Cumulative Incidence (95% CI)
0 months	9922	0	0.0
3 months	9834	27	0.3 (0.2–0.4)
6 months	9289	168	2.0 (1.7–2.3)
12 months	5348	1139	16.8 (15.9–17.6)
15 months	3693	615	27.3 (26.2–28.3)
18 months	2622	259	33.0 (31.8–34.1)
21 months	1606	346	43.6 (42.2–45.1)
24 months	1168	164	50.0 (48.4–51.6)

## Discussion

In this retrospective evaluation of VL testing frequency in Côte d’Ivoire, we found that most PLHIV received their first VL test within 12 months of ART initiation but did not receive subsequent VL monitoring tests within the recommended time frame of 6–12 months, depending on VL test results at the patient’s first test. A majority of the study participants were adults, aligning with the population of PLHIV in Côte d’Ivoire [3]. The study population was predominantly female and likely reflects gender differences in knowing one’s HIV status. A 2018 household-based national survey found that Ivorian women were more likely to know their HIV status, be on treatment, and be virally suppressed than their male counterparts; just 4 out of 10 men knew they were living with HIV [13]. At the time of this study, there was no national guidance on VL testing specifically for pregnant women, however it is possible that some of this difference is attributable to HIV integration in maternal and child healthcare. During antenatal care, women have more frequent interactions with the healthcare system and as a result may have increased opportunity for VL testing. Efforts should be made in Côte d’Ivoire to test men who may be living with HIV but unaware of their status and initiate them on ART. Until more PLHIV are aware of their HIV status and linked on ART, it will remain a challenge to assess VLS on a national scale.

Our results showed that a majority (79.6%) of patients were virally suppressed at their first VL test. These results are promising as they suggest high retention and ART efficacy within 6–12 months of ART initiation among the study population. However, when disaggregating by age, we found that children aged 0–14 years old were significantly less likely to be virally suppressed at their first test. While ART coverage among children living with HIV in Côte d’Ivoire is consistently high, VLS is lower than it is for adults, especially for children under five. This may in part be associated with the requirement for early infant diagnosis (EID) results to be received as a prerequisite to ART initiation. EID has many benefits including early ART initiation and is associated with reduced morbidity and mortality, however in resource-limited settings, loss to follow up during the EID care cascade and long delays in lab testing are common, contributing to delays in ART initiation and VL testing [14]. These findings are consistent with studies conducted in other settings in sub-Saharan Africa [15–17]. Low rates of VLS among children have also been attributed to limited availability of optimal pediatric ART regimens, potential HIV drug resistance from prevention of mother to child transmission (PMTCT) treatment exposure, and dependence on caregivers for retention [15]. Although this is not unique to our study context, it remains an important finding and highlights the need for innovative interventions to optimize ART treatment among children and support adherence to increase VLS rates. This finding also highlights the importance for programs to disaggregate VLS data into finer age categories to better understand and target interventions where improvements are needed.

While the median time to the first VL test was only slightly greater than the recommended 6-month interval, 25% of patients did not have their first VL test until more than a year after ART initiation. The estimated time to first VL test did not differ significantly by age or gender, but among patients who initiated ART in more recent years, time to first VL test shortened to align more with national recommendations. This is a promising trend, suggesting that provision of VL testing within 6 months of ART initiation is increasing over time.

Less than one third (29.7%) of the study population received a second VL test within the 4-year study period. Unlike the estimated time between ART initiation and first VL test, the time between first and second tests was 2–3 times the recommended 6-to-12-month interval. This did not differ significantly by age or gender, suggesting it is a widespread issue impacting all PLHIV. We estimated that the time interval between first and second VL tests was 3 months longer among patients who were not virally suppressed at their first VL test, despite recommendations for a shorter 6-month interval. However, for both groups, the time between tests was substantially greater than guidelines recommend. These findings have been observed in other contexts such as South Africa and Lesotho and support the need for strengthened, targeted efforts to reach virally unsuppressed patients for routine testing and potential ART regimen change [18, 19].

Routine VL monitoring can be a cost-effective and impactful approach to reaching and maintaining the 3^rd^ 95 UNAIDS goal: that 95% of patients who are retained on ART treatment are virally suppressed. However, cost-effectiveness is largely dependent on ensuring that results are acted upon in a timely manner for patients who are virally unsuppressed and used to simplify HIV care for patients who are virally suppressed [20]. We identified a need to strengthen targeted efforts to reach virally unsuppressed patients for timely routine testing and immediate ART regimen change. The findings from our study align with previous research suggesting that VL monitoring is often not done according to national or WHO guidelines for testing within 6- to 12-months depending on previous suppression results, thus reducing the benefits of VL monitoring [18, 19]. Lack of routine VL monitoring can lead to unnecessary ART regimen switches which in turn increase treatment costs, exhaust drug options, and can contribute to higher prevalence of ART drug resistance [21].

Recommendations to improve VL monitoring include healthcare worker trainings to highlight the importance of routine VL testing for all PLHIV and shorter testing intervals for unsuppressed PLHIV [4]. Evidence-based strategies to improve VL monitoring and increase suppression rates may include differentiated service delivery models (DSD) such as multi-month ART scripting to simplify HIV services, adherence clubs, and undetectable = untransmittable (U = U) communication strategies to encourage retention and knowledge of one’s own VLS status. Viral load champions, healthcare workers who monitor patient VL testing and suppression, should be employed and supported to improve timely testing intervals [22–25].

Our study had several strengths. Our data is not fully generalizable at the national scale considering data was used from laboratories based in the northeastern regions However, we assessed VL testing across several large regions in Côte d’Ivoire over four years using national public health data collected among patients attending both public and private health facilities. Data captured and extracted from OE allowed for precise turnaround time calculations and other testing metrics to be analyzed relatively quickly compared to paper-based methods. As such, we were able to study whether time between treatment, first VL test, and second VL test aligned with national recommendations, which has not been well categorized in previous research in Côte d’Ivoire or other low- and middle-income countries (LMICs). We found that OE is an effective tool to assess VLS and the quality of ART. Other national public health systems should attempt to utilize OE or other free, open-source laboratory information systems to monitor and evaluate VL testing and suppression at a national level and evaluate adherence to VL testing guidelines.

Our findings should be interpreted bearing in mind a few limitations. First, we only collected data from patients who were initiated on treatment and received a VL test. We expect that the true rate of VLS is lower among the total population of PLHIV in Côte d’Ivoire as not all are actively receiving ART treatment. We were unable to assess whether women in the study population were pregnant or breastfeeding, for whom VL testing guidelines differ. There is some potential that this artificially decreased the amount of time between tests slightly, as pregnant and breastfeeding women may have shorter intervals between VL testing. We were unable to analyze specific ART regimen or risk factors among the population. Due to the scope of this study, we did not disaggregate survival estimates of time between tests by age; however, children are recommended to receive VL testing every 6 months. Future research should analyze time between VL tests specifically among children and adolescents using more granular age categories.

We found that many patients in the study population had missing data for ART start date with only the year entered, and accuracy of the ART initiation date variable worsened over time. We were able to estimate the time to first VL test using an interval censoring approach to account for partial dates, which has been well validated for survival analyses [26, 27]. However, it is important to emphasize and advocate for accurate data entry to ensure patients are receiving high quality care, including timely VL tests, and to facilitate monitoring and evaluation of HIV programs. To address missingness in the data, we recommend implementing quarterly data quality assessments and annual training for laboratory staff and healthcare workers to highlight the importance of capturing accurate ART initiation data to improve adherence to guidelines for viral load monitoring. Configuring OE with data validation rules to ensure ART initiation date is non-missing and after date of birth should also be considered. In more recent years, OE has been optimized to strengthen the lab-clinic interface. This includes early-stage work linking OE with Côte d’Ivoire’s HIV electronic medical records (EMR) system, which will have positive implications on data quality, patient monitoring, and quality of care [28].

Our study highlights the importance of monitoring VL after initiation, especially among children and PLHIV who are unsuppressed at their first test. Results from this study suggest there are bottlenecks or challenges to VL monitoring which may allow the Côte d’Ivoire MSHP to prepare and train health care workers in the promotion of timely VL monitoring. This study also contributes to existing evidence that children are at highest risk of unsuppressed VL–the MSHP or implementing partners working in Côte d’Ivoire may consider differentiated service delivery (DSD) approaches to ART linkage and retention services for children living with HIV as an approach to increase VLS rates. Our study did not assess specific health system and workforce challenges, including breakdown of laboratory equipment, stockouts of VL testing supplies encountered by health care workers in laboratories and health facilities, or the type of facility and its funding mechanism (e.g., public or private). Future research should examine these system- and patient-level factors and their impact on routine VL monitoring.

Data in this study was collected before the onset of the COVID-19 pandemic, which had heterogeneous impacts on healthcare access and outcomes across Sub-Saharan Africa [29]. During the early stages of the COVID-19 pandemic, multi-month dispensing, a form of differentiated service delivery, was quickly adopted for clinically stable patients [30]. This has been associated with ART retention and VL suppression [30, 31]. However, due to reduced access to healthcare services stemming from the pandemic, VL monitoring may have been reduced significantly. In recent years, national investments have been made to prioritize increasing VL suppression among PLHIV. For example, in 2019, Côte d’Ivoire introduced Dolutegravir (DTG)-based regimen as the preferred first line ART [32] which has proven to be effective in helping patients attain VL suppression [33]. Furthermore, investments have been made to closely monitor patients and intensify adherence support [34]. As a result of these efforts, VL suppression rates have increased nationally [34].

In 2019, HIV and AIDS was the 4^th^ highest cause of death and disability in Côte d’Ivoire compared to 11^th^ globally [35, 36]. As such, VL monitoring in Côte d’Ivoire needs to be better utilized to its full potential, especially for populations at higher risk such as children, men, and virally unsuppressed PLHIV, it can help improve treatment outcomes by providing health care workers with the necessary tools to identify patients in need of enhanced treatment and retention support, ultimately preventing transmission and enabling PLHIV to live long and healthy lives. This research significantly contributes to scientific evidence from Côte d’Ivoire and West Africa, an understudied region, as most HIV and VL research has been published from countries in East and Southern Africa.

## Conclusions

In this evaluation of VL testing and suppression, we found VLS was lowest among children under the age of 15. While the estimated time for first VL test after ART initiation was aligned with national recommendations, timing for a subsequent VL test was significantly delayed. Our findings highlight the importance of regular VL monitoring after the first test, especially for children. Routine VL testing in accordance with national recommendations is imperative to reaching UNAIDS 95-95-95 goals.
